# Video-assisted thoracoscopic surgery versus thoracotomy for locally advanced lung cancers: A systematic review and meta-analysis

**DOI:** 10.1097/MD.0000000000047359

**Published:** 2026-01-23

**Authors:** Li Yanlin, Liu Ying, Lu Ya, Lin Dong

**Affiliations:** a Department of thoracic and cardiovascular surgery, Deyang People’s Hospital, Deyang City, Sichuan, China; b Department of urology, Pengzhou People’s Hospital, Pengzhou City, Chengdu, Sichuan, China.

**Keywords:** locally advanced lung cancers, meta-analysis, thoracotomy, Video-assisted thoracoscopic surgery

## Abstract

**Background:**

Locally advanced lung cancer is considered a relative contraindication for video-assisted thoracoscopic surgery (VATS), and VATS versus conventional open thoracotomy for locally advanced lung cancer has not been studied.

**Methods:**

We have searched the Embase, Cochrane Library, PubMed, Medline, and Web of Science for articles on laparoscopic and conventional open thoracotomy. We calculated pooled standard mean difference (SMD), relative risk, and 95% confidence intervals (CIs). The protocol for this review has been registered on PROSPERO (CRD420251240690).

**Results:**

There are 1597 participants including 11 articles. Compared with open thoracotomy, patients who underwent VATS had less operation time (SMD 0.15; CI 0.01–0.29; I^2^ = 0%, *P* = .031), blood loss (SMD −0.55; CI −0.69 to 0.42; I^2^ = 96.8%, *P* = .000), chest tube duration (SMD −0.17; CI −0.29 to −0.04; I^2^ = 38.3%, *P* = .009), and hospital stay (SMD −0.68; CI −0.86 to −0.49; I^2^ = 43.3%, *P* = .000). However, there are no difference in ICU days, lymph node resected, lymph node total stations, complications, acute respiratory distress syndrome, atrial arrhythmia, chylothorax, prolonged air leak, pneumonia, and overall recurrence.

**Conclusion:**

Patients who underwent VATS had less operation time, blood loss, chest tube duration, and hospital stay, compared with thoracotomy. There was no difference in complications, lymph node dissection, and overall recurrence between the 2 groups. More high-quality literature is needed to be included in the research in the future.

## 1. Introduction

Traditionally, patients with lung cancer undergo thoracotomy, which requires an incision of about 20 cm in the lateral chest wall, and even requires rib disconnection, which is traumatic and slow to recover, affecting the quality of life of patients.^[1]^ With the proposal of the concept of minimally invasive surgery, video-assisted thoracoscopic surgery (VATS) for lung cancer has developed rapidly. VATS has many advantages, such as less trauma, less postoperative pain, less lung function damage and faster recovery than thoracotomy, and has been widely carried out.^[2]^ With the development of VATS, many difficult problems have been solved.^[3]^ Studies have shown that compared with traditional thoracotomy, VATS has the same number of lymph node dissections, but there are significant differences in intraoperative blood loss, operation time, thoracic drainage volume, postoperative hospital stay, etc, showing the superiority of VATS.^[4,5]^ For patients with locally advanced lung cancer, traditional thoracotomy is often difficult to provide satisfactory treatment effect. The development of thoracoscopic surgery has provided new treatment options for these patients, especially for those who are not suitable for traditional thoracotomy.^[6,7]^ Therefore, it is necessary to study the meta-analysis of video-assisted thoracoscopic surgery versus traditional thoracotomy.

## 2. Methods

### 2.1. Protocol and guidance

The study had been performed according to Preferred Reporting Items for Systematic Reviews and the meta-analysis (PRISMA)^[8]^ and the quality evaluation of this article was scored using the Newcastle-Ottawa Scale (NOS) score. The protocol for this review has been registered on PROSPERO (CRD420251240690).

### 2.2. Search strategy

This study involved literature published in the Embase, PubMed, Cochrane Library, Medline, and Web of Science up to May 10, 2025. We defined the eligibility criteria according to the population (P), intervention (I), comparator (C), outcome (O), and study design approach.

P: The patients with locally advanced lung cancers.

I: undergoing thoracoscopic segmentectomy or lobectomy.

C: thoracotomy was performed as a comparator.

O: one or more of the following outcomes: operation time, blood loss, chest tube duration, ICU days, hospital stay, lymph node resected, lymph node total stations, complications, ARDS, atrial arrhythmia, chylothorax, prolonged air leak, pneumonia, and overall recurrence.

The MeSH terms “locally advanced lung cancer or lung neoplasm,” “thoracotomy or thoracotomy,” and “video-assisted thoracic surgery or VATS” and comparative study were used. The search strategy was not limited by language or year. It was not requested by the ethics or institutional review committee due to the study being designed as a systematic review and meta-analysis.

### 2.3. Inclusion and exclusion criteria

We have included the literature by the following criteria. Comparative data were available on the treatment of locally advanced lung cancer through minimally invasive and conventional thoracotomy. Outcome indexes should include at least one of the following, perioperative period, postoperative, and oncologic outcomes. Any study which did not confirm the above inclusion criteria was excluded.

### 2.4. Data extraction and outcome measures

Two researchers (L.Y. and L.D.) independently reviewed the retrieved literature by the inclusion and exclusion criteria. The third researcher (L.H.) was asked to participate in the discussion to decide whether to include when disagreements were encountered. The extracted data included the first author, publication, country, study type, group, age, clinical stage, and outcomes (if mentioned) (Table 1).

**Table 1:** The main characteristics of included studies.

Author	Publication	Country	Study period	Study design	Group	Cases	Age	Male (%)	Tumor size	Outcomes	Confounders adjustment	NOS score (max:9)
Chen et al. 2017^[9]^	J Thorac Cardiovasc Surg	China	since 2010	Prospective	VATS	250	62.2 ± 10.3	62.4	3.3 ± 1.6	②③⑤⑥⑦⑨⑩⑪⑫⑬⑭	No	8
					Open	161	60.2 ± 9.6	75.2	4.8 ± 2.2			
Fan et al. 2016^[10]^	Journal of thoracic disease	China	2013–2015	Retrospective	VATS	64	59.75 ± 10.5	78.8	4.8 (1–10)	②③⑤⑦⑧⑬⑭	No	7
					Open	68	59.78 ± 8.4	70.6	5.4 (1.8–10)			
Fang et al. 2018^[11]^	J Cardiothorac Surg	China	2013–2017	Retrospective	VATS	14	61 (55–73)	78.6	2.5 (1.0–7.0)	③⑥⑨⑩⑭	No	7
					Open	67	60 (29–77)	94	3.1 (0.8–8.0)			
Hennon et al. 2011^[12]^	Ann Surg Oncol	USA	2002–2007	Retrospective	VATS	95	68.6 (42.4–85.9)	52.6		②③④⑥⑦⑧⑫⑬⑭	No	8
					Open	19	67.0 (47.8–83.7)	47.4				
Hireche et al. 2023^[13]^	Cancers (Basel)	France	2013–2020	Retrospective	VATS	64	62.03 ± 8.03	56.2	42.3 ± 30	①②⑥⑦⑨⑩⑪⑫⑬⑭	Yes (propensity score matching)	8
					Open	64	63.09 ± 9.9	59.3	31.4 ± 18.9			
Jeon et al. 2018^[14]^	Annals of Surgery	Korea	2009–2013	Retrospective	VATS	17	62.7 ± 7.9	82	26.3 ± 12.9	①⑦⑨⑫⑬	No	8
					Open	18	60 ± 8.7	95	40.6 ± 31.9			
Pan et al. 2023^[15]^	Thoracic cancer	China	2016–2021	Retrospective	VATS	14	56.62 ± 10.72	78.57	37.07 ± 8.53	①⑥⑧⑨⑩⑪⑬⑭	No	7
					Open	23	56.74 ± 10.36	69.57	39.57 ± 11.49			
Pan et al. 2024^[16]^	Clin Lung Cancer	China	2019–2023	Retrospective	VATS	62	61.8 ± 8.1	87.1	3.6 ± 1.2	②③⑤⑥⑫⑭	No	8
					Open	62	61.0 ± 6.9	90.3	3.7 ± 1.4			
Wei et al. 2021^[7]^	Annals of surgical oncology	China	2017–2019	Prospective	VATS	63	58.04 (49.18–64.15)	71.43		④⑦⑭	No	8
					Open	54	57.96 (49.49–64.90)	81.48				
Yang et al. 2016^[17]^	Eur J Cardiothorac Surg	USA	1996–2012	Retrospective	VATS	30	61.6 ± 11.4	60		⑦⑫⑬	Yes (propensity score matching)	8
					Open	30	60.7 ± 8.9	57				
Zhou et al. 2013^[18]^	Thorac Cancer	China	2004–2008	Prospective	VATS	117	58.21 ± 11.00	59		②③⑤⑥⑭	No	7
					Open	241	58.64 ± 11.18	64.7				

Matching: 1 - Age; 2 - BMI; 3 - Tumor size. NA, data not available. NOS score: Newcastle-Ottawa Scale score.①: ARDS; ②: Atrial arrhythmia; ③: Blood loss; ④: Chest tube duration; ⑤: Chylothorax; ⑥: Complications; ⑦: Hospital stay; ⑧: ICU, days; ⑨: Lymph node resected; ⑩: Lymph node total stations; ⑪: Overall recurrence; ⑫: Pneumonia; ⑬: Prolonged air leak; ⑭: surgery.

### 2.5. Statistical analysis

Statistical analysis was performed by Stata v.12.0 (Stata Corp LLC, College Station). For this meta-analysis, if the heterogeneity test was I^2^ > 50%, *P* < .1, we used the random effect model; if the heterogeneity test was I^2^ < 50%, *P* > .1, we used the fixed utility model. The combined r values and 95% confidence intervals (CIs) of each study were calculated, and the forest map displayed the characteristics of each study result. The quality of the included literature was evaluated using the NOS. The Begg and Egger tests were used to test the publication bias. The *P* < .05 was indicated as statistically significant.

## 3. Results

### 3.1. Eligible studies and study characteristics

**Figure 1.**
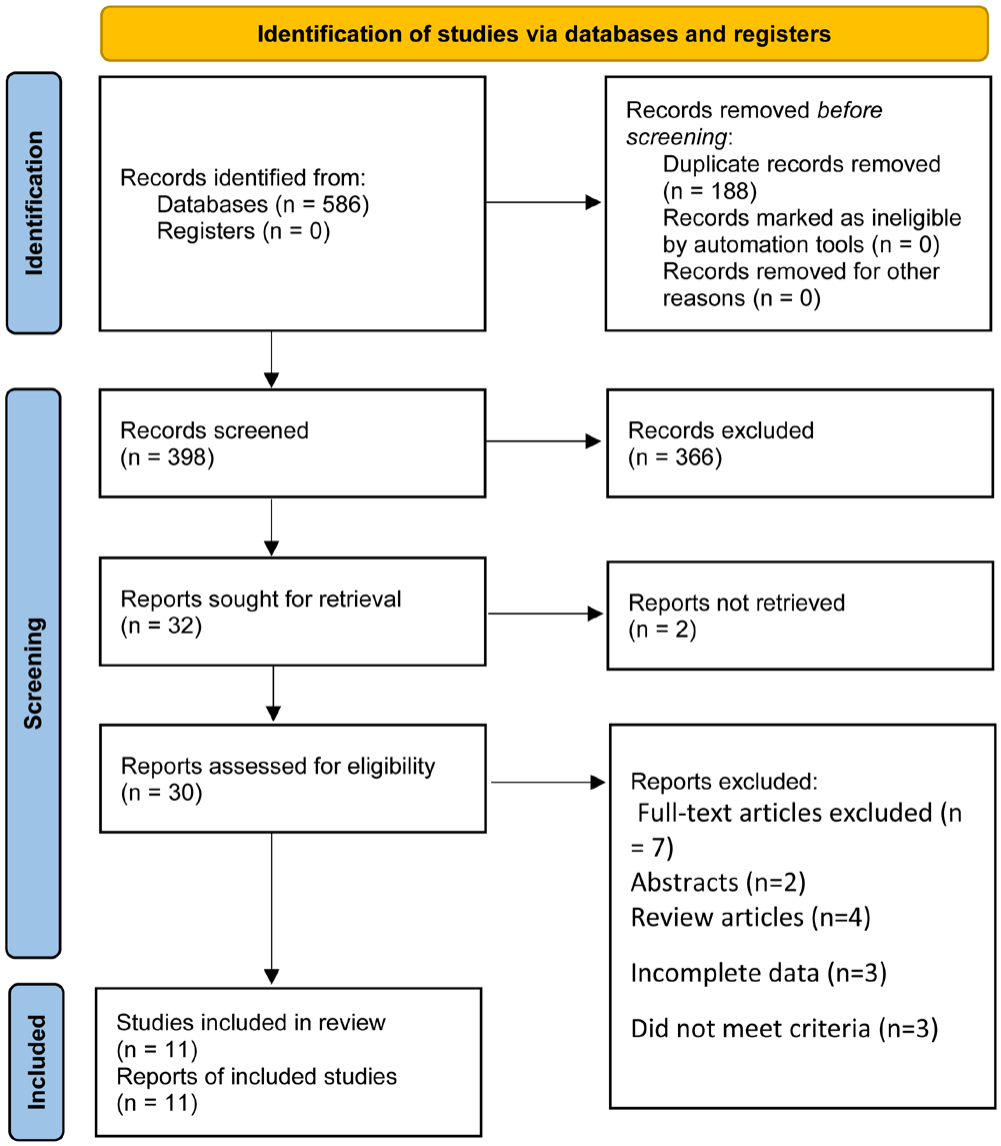
Flowchart for records selection process of the meta-analysis. (According to PRISMA template: Moher D, Liberati A, Tetzlaff J, Altman DG, The PRISMA Group (2009). Preferred Reporting Items for Systematic Reviews and Meta-Analyses: The PRISMA Statement. PLoS Med 6(7): e1000097. doi:10.1371/journal. Pmed 1000097).

We initially searched 586 records. 188 literature that was published repeatedly and cross-published were deleted. After reading the title and abstract, 366 articles were excluded. After the remaining 32 pieces of literature were searched for full text, reading, and quality assessment, 11 pieces of literature^[7,9–18]^ (1597 participants: VATS: 790 vs thoracotomy: 807) were eventually included (Fig. 1: Guidelines Flow Diagram). The detailed information on this literature is listed in Table 1.

### 3.2. Outcomes

#### 3.2.1. Perioperative outcomes

**Figure 2.**
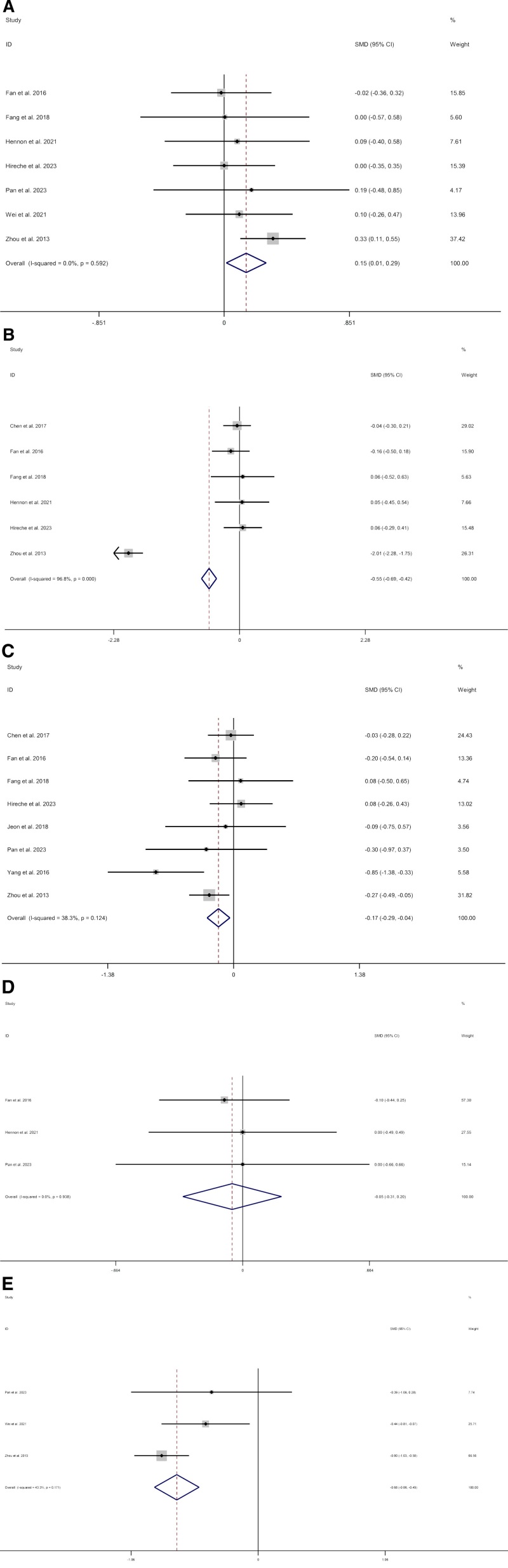
Meta-analysis of minimally invasive surgery versus thoracotomy for locally advanced lung cancers: (A) operation time, (B) blood loss, (C) chest tube duration, (D) ICU days, (E) hospital stay.

We included 7 studies^[7,10–13,15,18]^ about operation time. Compared with thoracotomy, patients who underwent VATS had less operation time (SMD 0.15; CI 0.01 to 0.29; I^2^ = 0%, *P* = .031) (Fig. 2A). Data on blood loss were reported in 6 studies.^[9–13]^ Compared with thoracotomy, patients who underwent VATS had less blood loss (SMD −0.55; CI −0.69 to 0.42; I^2^ = 96.8%, *P* = .000). Owing to high heterogeneity (I^2^ = 96.8%), sensitivity analysis cannot reduce heterogeneity. Therefore, we choose random effect model results (SMD −0.55; CI −0.69 to 0.42; I^2^ = 96.8%, *P* = .000) (Fig. 2B). Data on chest tube duration were reported in 8 studies.^[9–11,13–15,17,18]^ Compared with thoracotomy, patients who underwent VATS had less chest tube duration (SMD −0.17; CI −0.29 to −0.04; I^2^ = 38.3%, *P* = .009) (Fig. 2C). We included 3 studies^[10,12,15]^ about ICU days. Compared with thoracotomy, patients who underwent VATS had no difference (SMD −0.05; CI −0.31 to 0.20; I^2^ = 0%, *P* = .678) (Fig. 2D). Data on hospital stay were reported in 4 studies.^[7,11,15,18]^ Compared with thoracotomy, patients who underwent VATS had less hospital stay (SMD −0.61; CI −0.78 to −0.43; I^2^ = 68.8%, *P* = .000). Owing to high heterogeneity (I^2^ = 68.8%), sensitivity analysis was carried out by Stata 12.0. After removing the studies by Fang et al^[11]^ as the sample that was “left out,” the pooled results did not change substantially but the heterogeneity was significantly reduced (SMD −0.68; CI −0.86 to −0.49; I^2^ = 43.3%, *P* = .000) (Fig. 2E).

**Figure 3.**
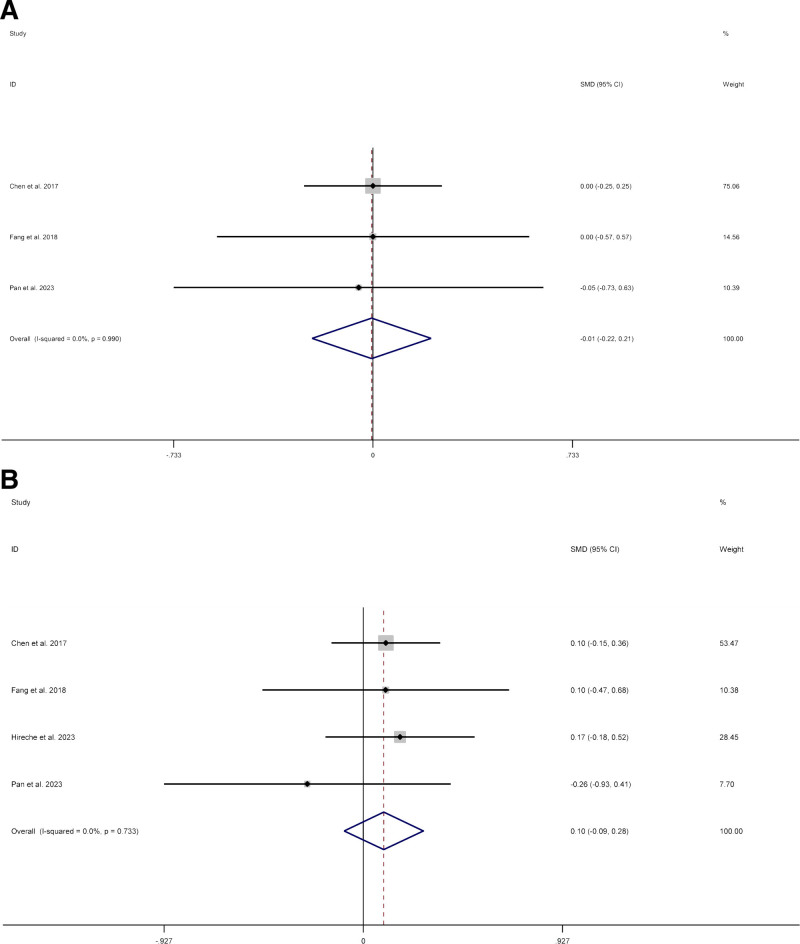
Meta-analysis of minimally invasive surgery versus thoracotomy for locally advanced lung cancers: (A) lymph node total stations, (B) lymph node total stations.

We included 4 studies^[9,11,13,15]^ about lymph node total stations. Compared with thoracotomy, patients who underwent VATS had no difference (SMD 0.18; CI −0.01 to 0.37; I^2^ = 69.8%, *P* = .06). Owing to high heterogeneity (I^2^ = 69.8%), sensitivity analysis was carried out by Stata 12.0. After removing the studies by Hireche et al^[13]^ as the sample that was “left out,” the pooled results did not change substantially but the heterogeneity was significantly reduced (SMD −0.01; CI −0.22 to 0.21; I^2^ = 0%, *P* = .961) (Fig. 3A). Data on lymph node resected were reported in 5 studies.^[9,11,13–15]^ Compared with thoracotomy, patients who underwent VATS had no difference (SMD 0.16; CI −0.02 to 0.34; I^2^ = 58.4%, *P* = .074). Owing to high heterogeneity (I^2^ = 58.4%), sensitivity analysis was carried out by Stata 12.0. After removing the studies by Jeon et al^[14]^ as the sample that was “left out,” the pooled results did not change substantially but the heterogeneity was significantly reduced (SMD 0.10; CI −0.09 to 0.28; I^2^ = 0%, *P* = .310) (Fig. 3B).

**Figure 4.**
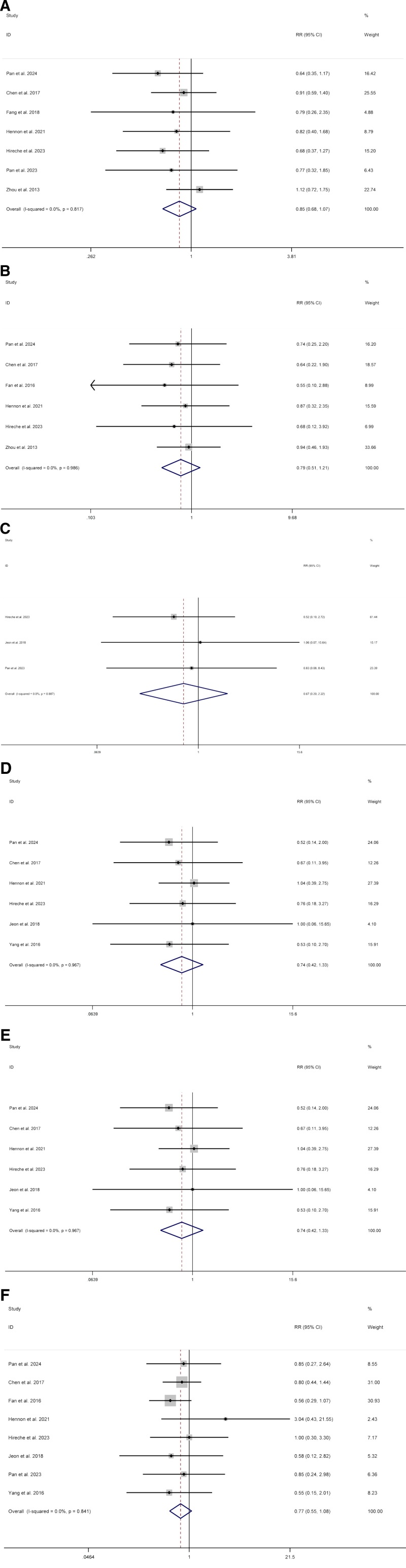
Meta-analysis of minimally invasive surgery versus thoracotomy for locally advanced lung cancers: (A) complications, (B) atrial arrhythmia, (C) acute respiratory distress syndrome (ARDS), (D) chylothorax, (E) pneumonia, (F) prolonged air leak.

We included 7 studies^[9,11–13,15,16,18]^ about complications. Compared with thoracotomy, patients who underwent VATS had no difference (RR 0.85; CI 0.68–1.07; I^2^ = 0%, *P* = .171) (Fig. 4A). Data on atrial arrhythmia were reported in 6 studies.^[9,10,12,13,16,18]^ Compared with thoracotomy, patients who underwent VATS had no difference (RR 0.79; CI 0.51–1.21; I^2^ = 0%, *P* = .279) (Fig. 4B). We included 3 studies^[13–15]^ about ARDS. Compared with thoracotomy, patients who underwent VATS had no difference (RR 0.67; CI 0.20–2.22; I^2^ = 0%, *P* = .514) (Fig. 4C). Data on chylothorax were reported in 4 studies.^[9,10,16,18]^ Compared with thoracotomy, patients who underwent VATS had no difference (RR 1.13; CI 0.47–2.71; I^2^ = 0%, *P* = .783) (Fig. 4D). Data on pneumonia were reported in 6 studies.^[9,12–14,16,17]^ Compared with thoracotomy, patients who underwent VATS had no difference (RR 0.77; CI 0.55–1.08; I^2^ = 0%, *P* = .316) (Fig. 4E). We included 8 studies^[9,10,12–17]^ about prolonged air leak. Compared with thoracotomy, patients who underwent VATS had no difference (RR 0.77; CI 0.55–1.08; I^2^ = 0%, *P* = .128) (Fig. 4F).

#### 3.2.2. Oncology outcomes

**Figure 5.**
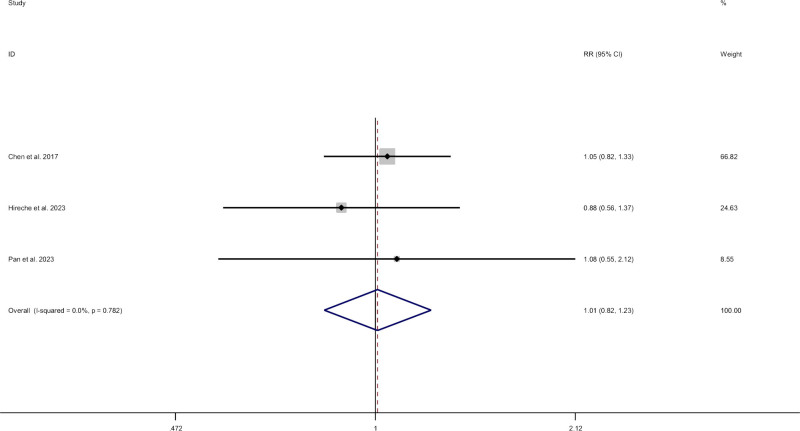
Meta-analysis of minimally invasive surgery versus thoracotomy for locally advanced lung cancers in overall recurrence.

Data on overall recurrence were reported in 3 studies.^[9,13,15]^ Compared with thoracotomy, patients who underwent VATS had no difference (RR 1.01; CI 0.82–1.23; I^2^ = 0%, *P* = .939) (Fig. 5).

## 4. Publication bias

We have conducted publication bias on more than 10 included studies by Begg test. Therefore, no publication bias is made here.

## 5. Discussion

N2 (IIIA) stage non-small cell lung cancer is a heterogeneous group of diseases, and there is still some controversy about the treatment of this group of diseases.^[19]^ Albain et al^[20]^ enrolled 429 patients with stage Ⅲ a NSCLC and randomly compared surgery with radical radiotherapy after concurrent chemoradiotherapy. The results showed that the OS of the 2 groups was similar, but the surgery group had a certain PFS advantage, and subgroup analysis showed that patients who underwent lobectomy had an OS advantage, while patients who underwent pneumonectomy had no OS benefit. Based on this, surgery has also become one of the treatment strategies for IIIA non-small cell lung cancer. With the increasing importance of surgery in N2 non-small cell lung cancer. In the past, traditional open surgery was used for such patients. With the development of minimally invasive surgery in recent years, the use of minimally invasive surgery for locally advanced lung cancer has become more and more widely.^[21,22]^

In the past, traditional thoracotomy was mainly used to treat locally advanced lung cancer. Traditional thoracotomy has many disadvantages, such as large trauma, more intraoperative bleeding, more postoperative complications, and long hospital stay, which brings great physical and psychological pain to patients.^[16,18]^ Compared with these shortcomings of traditional thoracotomy, thoracoscopic technology shows great advantages, with less trauma, less bleeding, beautiful appearance and fast recovery. It is favored by most patients, making more and more doctors committed to the development of minimally invasive technology. With the continuous development of video-assisted thoracoscopic technology, compared with traditional thoracotomy, video-assisted thoracoscopic technology has less trauma and faster postoperative recovery. For patients who need postoperative adjuvant treatment, it can accept postoperative comprehensive treatment more quickly. However, at present, video-assisted thoracoscopic technology is mainly used for early-stage non-small cell lung cancer (stage I-II).^[23,24]^ For locally advanced lung cancer, traditional thoracotomy is still the main treatment. With the progress of surgical treatment technology for lung cancer, the curative effect of surgery based comprehensive treatment for patients with partially resectable locally advanced lung cancer (stage III) is positive.^[25,26]^ Lung cancer invading the root of pulmonary vein and left atrium has been stage IIIB (T4). It is locally advanced lung cancer, and the possibility of distant metastasis is very high. The surgical treatment is very traumatic, and it is extremely difficult to complete the operation. Therefore, the indications should be carefully selected, especially when the tumor invades multiple organs, such as esophagus, tracheal carina, aorta, pulmonary artery, pulmonary vein and atrium, or when the tumor widely invades mediastinal lymph nodes and cannot be completely removed, the prognosis is poor.^[27,28]^ Although literatures^[7,9]^ suggested that there is no difference in less time, blood loss, chest tube duration, and hospital stay between VATS and thoracotomy. Our study shows that patients who underwent VATS had less operation time, blood loss, chest tube duration, and hospital stay, compared with thoracotomy. There was no difference in complications, lymph node dissection, and overall recurrence between the 2 groups.

Several limitations should be considered when interpreting the findings of this study. The analysis for certain outcome measures was constrained by a limited number of available studies, potentially affecting the robustness of those results. Furthermore, the current body of evidence consists predominantly of case-control designs, a number of which demonstrate only moderate methodological quality. The validity of long-term conclusions is also tempered by insufficient follow-up durations in some included studies, limiting the assessment of long-term outcomes. Consequently, future validation through large-scale, prospective studies with rigorous methodologies and extended follow-up periods is warranted to substantiate these findings.

## 6. Conclusion

Patients who underwent VATS had less operation time, blood loss, chest tube duration, and hospital stay, compared with thoracotomy. There was no difference in complications, lymph node dissection, and overall recurrence between the 2 groups. More high-quality literature is needed to be included in the research in the future.

## Author contributions

**Conceptualization:** Li Yanlin.

**Data curation:** Li Yanlin.

**Formal analysis:** Li Yanlin, Lu Ya.

**Funding acquisition:** Li Yanlin.

**Investigation:** Liu Ying, Lu Ya.

**Methodology:** Liu Ying.

**Project administration:** Liu Ying, Lu Ya.

**Resources:** Li Yanlin.

**Software:** Li Yanlin, Lu Ya.

**Supervision:** Lin Dong.

**Validation:** Lu Ya, Lin Dong.

**Visualization:** Lin Dong.

**Writing – original draft:** Lin Dong.

**Writing – review & editing:** Li Yanlin.
